# Betaine attenuate chronic restraint stress-induced changes in testicular damage and oxidative stress in male mice

**DOI:** 10.1186/s12958-022-00949-8

**Published:** 2022-05-21

**Authors:** Xingqi Meng, Lixuan Peng, Jie Xu, Dongming Guo, Wenyu Cao, Yang Xu, Suyun Li

**Affiliations:** 1 Clinical Anatomy & Reproductive Medicine Application Institute, Hengyang Medical School, University of South China, Hengyang, 421001 Hunan China; 2 Department of Physiology, Hengyang Medical School, University of South China, Hengyang, 421001 Hunan China

**Keywords:** Betaine, Chronic restraint stress, Spermatogenic dysfunction, Oxidative stress

## Abstract

**Scope:**

Male fertility and sperm quality are negatively affected by psychological stress. Chronic restraint stress (CRS) is a common psychological stress that has a negative effect on sperm. Betaine (BET), an active ingredient isolated from Lycium barbarum, has anti-oxidant, anti-inflammatory and other pharmacological activities. This study aims to explore whether betaine has a therapeutic effect on sperm deformity and vitality under CRS and its mechanism.

**Methods and results:**

Chronic restraint stress was induced in 8-week-old male C57BL/6 J mice by fixation for 6 h a day for 35 days. Mice were intraperitoneally injected with betaine (BET) or normal saline (NS) for 14 days. Thirty-five days later, the animals were sacrificed. The results showed that the detrimental effects of CRS on testes as evident by disrupted histoarchitecture, increased oxidative stress, inflammation and apoptosis that compromised male fertility. BET injections can reverse these symptoms.

**Conclusions:**

BET can improve spermatogenesis dysfunction caused by CRS, which may provide potential dietary guidance.

**Graphical abstract:**

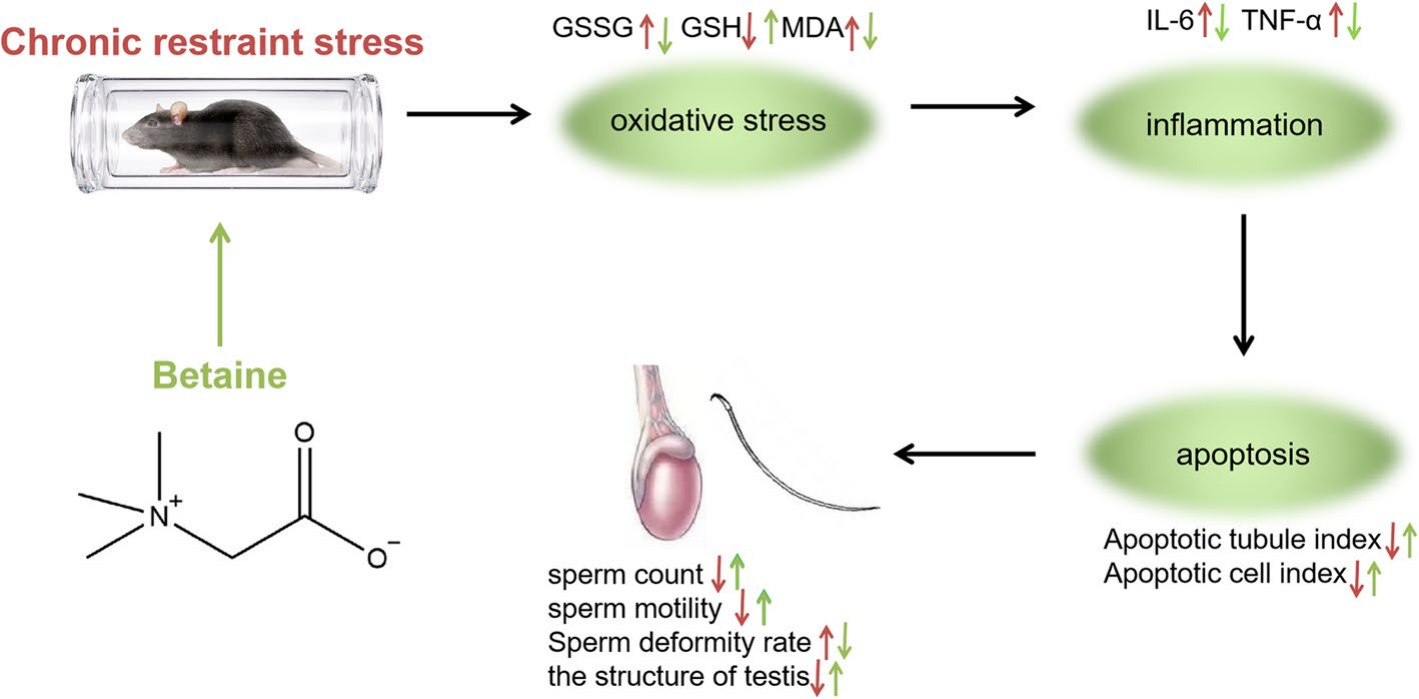

**Supplementary Information:**

The online version contains supplementary material available at 10.1186/s12958-022-00949-8.

## Introduction

Semen quality, indicator of male reproductive health and fertility, is closely related to human fertility. However, about 15% of couples of childbearing age have infertility problems, and about half of them might be due to the low quality of semen [1]. Multifactorial causes, such as failure in spermatogenesis, defects in sperm transportation, hormonal dysfunction, aging, and environment and lifestyle factors have been suggested to be involved in low quality of semen [2]. Among these factors, physiological and psychological stress seems to be one of the main causes of dysfunction of male reproductive health [3]. It has been demonstrated chronic stress results in an adverse effect on endocrine function and the male reproductive system, including alterations in androgenic hormone levels, sperm maturation, and testicular function [4]. Under chronic stress conditions, the oxidative and antioxidant systems of the reproductive system are out of balance, and the over-activation of oxidative stress triggers inflammation and destroys the microenvironment of spermatogenesis [5, 6]. A defect in testicular enzymatic antioxidants, including superoxide dismutase (SOD), catalase (CAT), and glutathione peroxidases (GSH-Px), as well as an increase in lipid peroxides measured as malondialdehyde (MDA), has been discovered in chronic stress-exposed animals [7]. Thus, effective treatment to re-balance the oxidative and antioxidant systems might improve the semen quality and protect the spermatogenesis [8].

Betaine (BET), also known as trimethylaminohydantoin, is widely found in animals, plants and microorganisms [9]. Betaine is generally supplemented through dietary intake, and 9–15 g per day is the appropriate intake for humans [10]. As an important methyl donor, BET catalyzes the formation of methionine from homocysteine in the liver and kidney [11]. Recent studies have shown that BET can play an important role in antioxidants, and protect the liver from oxidative stress [12, 13]. However, it is still unknown whether BET treatment could prevent the chronic stressed induced oxidative stress in mouse testis.

The aforementioned potential roles of BET led us to hypothesize that BET maybe antagonize oxidative stress injury and cell apoptosis of testis induced by chronic restraint stress, a validated stress model [6]. Thus, this study aimed to evaluate the effect of BET and its possible function with CRS-induced toxicity spermatogenic dysfunction in mouse testis. In addition, multiple test methods were assessed involvement of oxidative stress, anti-inflammatory and apoptosis on BET mediated protection against CRS-induced testicular toxicity. Our results will provide guidance for against reproductive toxicity caused by chronic stress.

## Materials and methods

### Animals

36 healthy, 8 weeks male C57BL/6 J mice that weighed 22–25 g (obtained from the Hunan SJA Lab Animal Center of Changsha, Hunan, China) was used in this study. Mice were group-housed (4–5 mice per cage) at a controlled temperature (22 ± 2℃) under a 12-h light/dark cycle with free access to food and water. The mice adapted to their new environment for 7 days before the behavioral tests. The experimental protocol was approved by the Animal Care and Use Committee of the University of South China (permit number: USC2020031602) and conformed to the National Institutes of Health Guide for the Care and Use of Laboratory Animals.

### Drugs dosage information and treatments

**Fig. 1:**
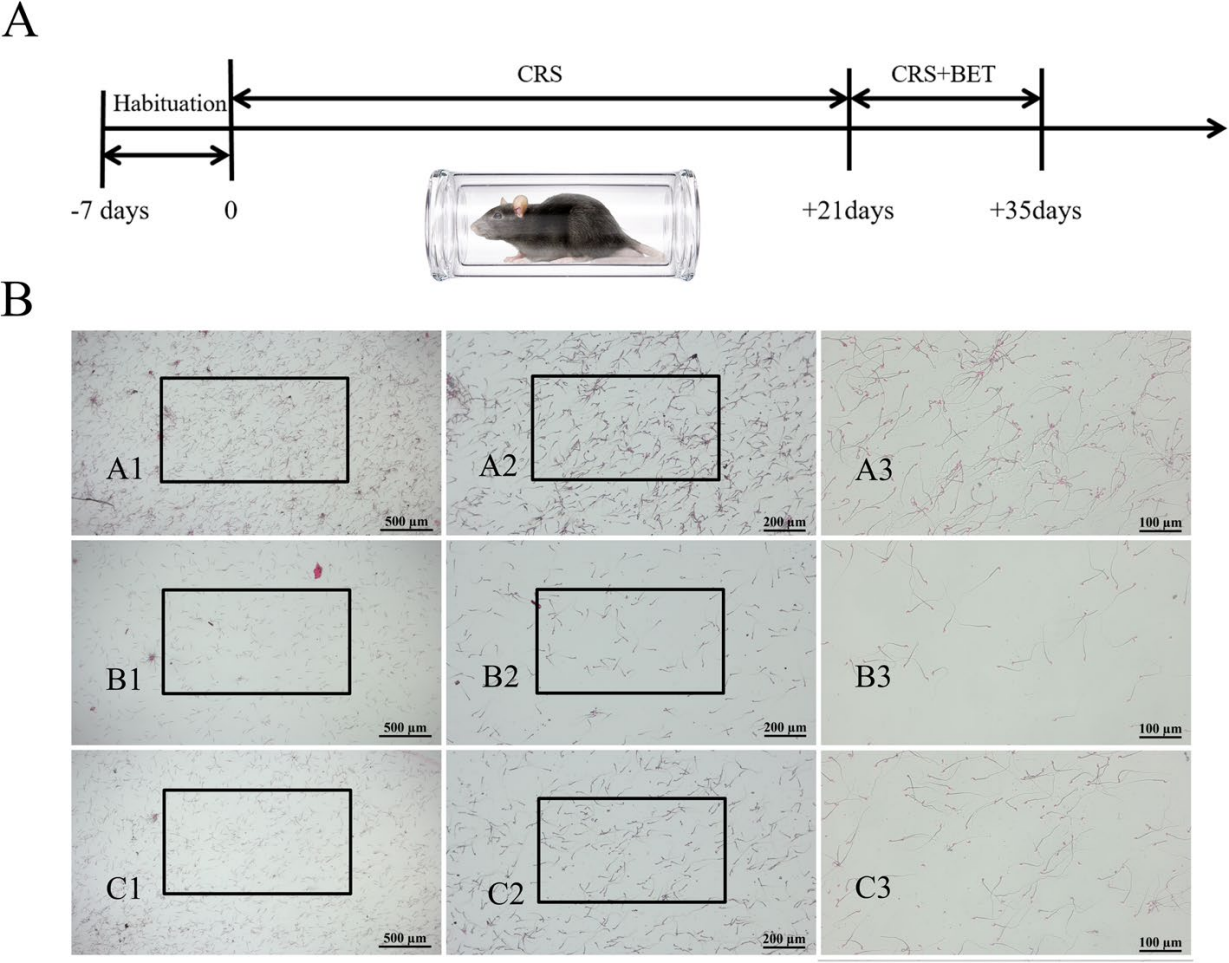
Effects of Betaine on organ and sperm parameters in chronic stressed mice. (**A**) Schema for chronic restraint stress (CRS) treatment schedule and sampling. BET Injection: give mice intraperitoneal injection of betaine, 200 mg/KG. (*n* = 12 male mice). (**B**) Effects of Betaine (BET) treatment on different forms of morphological abnormalities in spermatozoa. (eosin staining, observation under 50, 100, 200 magnification): (A1-A3) control group; (B1-B3)CRS group; (C1-C3) BET 200 mg/ kg treated group (*N* = 6 male mice)

After adaptive feeding for 7 days, the mice were randomly divided into Control + NS group, CRS + NS group, CRS + BET group. CRS + NS and CRS + BET groups' animals were kept in an acrylic cylinder (inner diameter 6.5 cm, length 20 cm, cylinder nose with ventilation holes) from 9:00–15:00, and the procedure lasts for 35 days. On the 21st day, the animals in the CRS + BET group were intraperitoneally injected with BET (Sigma, Dissolved in 0.9% NaCl) at a dose of 200 mg per kilogram of body weight everyday for 14 days [14], while animals in the CRS + NS group were injected with the same amount of NS. Body weight was measured every week. After sacrificed, the testes, epididymis and serum were taken out for further molecular mechanism testing. The experimental procedure was shown in Fig. 1A.

### Biochemical analysis of antioxidant status

Frozen testis tissue was homogenized in Homogenizing medium for 3 min to prepare a 10% (w/v) homogenate. The homogenates were filtered and centrifuged at 3500 rpm and 4 ℃ for 15 min using a refrigerated centrifuge and the activities of MDA (Solarbio, BC0025, China), GSSG (Elabscience, E-BC-K097-M, China) and GSH (Elabscience, E-BC-K030-M, China) were determined according to the instruction. The protein content of the supernatant was determined with the BCA assay kits (CWBIO, China). The assay results were normalized to the protein concentration in each sample, and were expressed as U mg^−1^ protein or nmol mg^−1^ protein.

### Sperm count analysis

Sperm analysis was conducted according to the method described in the published literature with a slight modification [15]. In brief, the right cauda epididymis was weighed and finely minced using anatomical scissors in 2 mL of pre-warmed physiological saline in a petri dish to allow the spermatozoa to swim freely out of the small nicks in the epididymides. After filtering with 200 nylon nets, the fluid was incubated at 37 ℃ for 5 min and then diluted in a ratio of 1:10. The number of sperm was counted by the traditional method using a hemocytometer under a light microscope at 400 magnification.

### Spermatozoa viability assessment

Approximately 20 μL of eosin dye liquor was mixed with an equal volume of spermatozoa suspension. After incubation at room temperature for 2 min, the slides were observed under a light microscope with 400 × magnification (Zeiss). Dead sperm appeared pink and live sperm were not stained. A total of one hundred sperm were counted in order to obtain the ratio of live to dead sperm [16].

### Morphological evaluation using light microscopy

The testicular tissues were fixed in Bouin’s fixative overnight at 4℃, dehydrated in alcohol gradient, cleaned in xylene and then embedded in paraffin blocks. The embedded testis tissue wax was cut into 5 μm slices, after the slices were unfolded in a 37℃ film expander, the slides were taken out, then, the water was dried and then packed into boxes. Paraffin sections were dewaxed with xylene and different concentrations of alcohol, and then stained with hematoxylin and eosin. The slides dyed by H&E, after being dehydrated and transparent, were sealed with neutral resin, and the microstructure was observed under optical microscope. Histopathological changes in the seminiferous tubules were observed using an ocular micrometer (Zeiss) at a magnification of 400 × and images were obtained [17]. Johnsen's mean testicular biopsy score (MTBS) criteria was used to evaluate the structure of the testis and the damage of sperm. Each group selects 6 mice's different cross-sections, and divides them into 0–10 points according to the maturity of the germinal epithelium of the seminiferous tubules. After single-blind scoring by different researchers, a systematic analysis was performed [18]. The Mean testicular biopsy score (MTBS) classifcation were as Table 1.

**Table 1:** Mean testicular biopsy score (MTBS) classifcation

Score	Description
1	No cells in tubular section
2	No germ cells but Sertoli cells are present
3	Spermatogonia are the only germ cells present
4	Only few spermatocytes and no spermatids or spermatozoa present
5	No spermatozoa,no spermatids but several or many spermatocytes present
6	No spermatozoa and only few spermatids present
7	No spermatozoa but many spermatids present
8	Only few spermatozoa present in section
9	Many spermatozoa present but germinal epithelium disorganized with marked sloughing or obliteration of lumen
10	Complete spermatogenesis with many spermatozoa. Germinalepithelium organized in a regular thickness leaving an open lumen

### RNA extraction and real-time PCR analysis

After being anesthetized with 10% sodium pentobarbital (45 mg/kg), the testis of mice were removed and frozen at − 80℃ until using. Total RNA was extracted by the Trizol® reagent (CWBIO), according to the manufacturer’s instructions. RNA purity was determined by A260 nm/A280 nm absorption ratio. Total RNA cDNA synthesis was performed with the RevertAidTM First Strand cDNA synthesis kit (Fermentas) according to the manufacturer’s instructions, using 2 μg of total RNA. And the cDNA was stored at -20℃. Expression levels of genes were determined by ABI-7500 real-time PCR system employing the TB GreenTM Premix Ex TaqTM II (Takara). The GAPDH gene expression was used as an internal control. The Primers were designed with Primer 3 software. A two-step PCR protocol was used according to the manufacturer’s instructions. PCR cycling conditions were as follows: 30 s at 95℃, followed by 40 cycles at 95℃ for 10 s and 60℃ for 30 s. Samples were processed in technical duplicates and a melting analysis was performed for each sample at the end of the PCR. 2^−△△Ct^ method was used to determine the relative gene expression according to our previous study [19]. The primer sequences were as Table 2.

**Table 2:** Primers used for PCR amplification

Primer sequence		
Gene	Forward	Reverse
IL-6	CTCCCAACAGACCTGTCTATAC	CCATTGCACAACTCTTTTCTCA
IL-1β	GCAGAGCACAAGCCTGTCTTCC	ACCTGTCTTGGCCGAGGACTAAG
TNF-α	ATGTCTCAGCCTCTTCTCATTC	GCTTGTCACTCGAATTTTGAGA
AR	TAAAGACATTTTGAACGAGGCC	GTCAGATATGGTTGAATTGCCC
CD29	TACTCTGGAAAATTCTGCGAGT	ATAGCATTCACAAACACGACAC
INSL3	CTAGAGCAGAGACATCTCCTG	CTGAGAAGCCTGGAGAGGAAG
GAPDH	ACCACCATGGAGAAGGCTGG	CTCAGTGTAGCCCAGGATGC

### Western blot analysis

Mice were anesthetized with an overdose of 10% sodium pentobarbital (45 mg/kg), and the testis of mice was rapidly removed following decapitation. Frozen testis tissue was homogenized in Tissue Protein Extraction plus Protease Inhibitor (CWBIO). Dissolved proteins were collected after centrifugation at 12,000 rpm for 20 min at 4℃, and supernatants were collected. Then, protein concentrations were determined using BCA Assay kit (CWBIO). Concentrations of 15–30 μg total protein were then separated by 10%-15% sodium dodecyl sulfate–polyacrylamide gel electrophoresis (SDS-PAGE) at 120 V for 1.5 h on gradient polyacrylamide gels. Membranes were blocked in 5% skim milk solution for 2 h, followed by incubation with primary antibodies (Table 3) for 2 h at room temperature and subsequent incubation overnight at 4℃. After being rinsed three times in 0.05% Tween-Tris buffered solution, membranes were incubated in goat anti-rabbit or goat anti-mouse conjugated horseradish peroxidase (HRP) secondary antibodies (1:1000, CWBIO) for 2 h followed by development with an enhanced chemiluminescence (ECL) system (CWBIO). β-Tubulin was used as a loading control. The optical density of each band was measured using the NIH program ImageJ (NIH, Bethesda, MD, USA) and was normalized to β-Tubulin.

**Table 3:** Primary antibodies used in this study

Antibody	Specificity	Type	Dilution	Source
β-Tubulin	Mouse	Polyclonal	1:10,000	Proteintech
AR	Rabbit	Polyclonal	1:1000	Proteintech
CD29	Rabbit	Polyclonal	1:1000	Abconal
GFRA1	Rabbit	Polyclonal	1:1000	Abconal
INSL3	Rabbit	Polyclonal	1:1000	Abconal

### Immunohistochemistry

Expression of INSL3 was detected using an IHC method. The PV-9000 kit was purchased from Beijing Zhongshan Golden Bridge Biotechnology Company. Rabbit Polyclonal antibody INSL3 was from the Abconal (working dilution 1:50). All procedures were implemented according to the manufacturer’s instructions. Paraffin sections of the testis were placed in xylene and deparaffinized by ethanol with different concentration gradients. Refer to the instructions for antigen retrieval and block endogenous peroxidase. Add the primary antibody dropwise and then add the reaction enhancement solution after incubation. DAB develops color after adding enhanced enzyme-labeled goat anti-mouse/rabbit IgG polymer. After counterstaining, mount the slides, observe and judge under an optical microscope. The analysis and semi-quantification of positive immunoreaction was downloaded from performed using the software Image-Pro Plus 6.0 software (Media Cybernetics Inc., USA) at a magnification of 400 × in microscope. Representative images were captured for each sample under the same light intensity and exposure time conditions. The mean-to-area ratio of the integrated optical density parameter (mean IOD) for each subject was used as semiquantitative [20].

### Apoptosis assessment using TUNEL assay

The sections were stained by TUNEL (DNTT dUTP gap end labeling) using in situ cell apoptosis assay kit (Boster Biotechnology, Wuhan, Hubei, China) according to the manufacturer's instructions. After dehydrated and encased in paraffin, the testes slices (5 μm) were immersed in xylene to dewax and rehydrate. The slices were added with 30 μl proteinase K working solution and incubated at 37℃ for 10 min. After rinsing with phosphate buffered saline (PBS, 10 mmol/L, pH = 7.5), the slices were added with 20 μl labeling Buffer and incubated at 37℃ for 2 h. After washing, the slices were added with 50 μl blocking Reagent and incubated at room temperature for 30 min. After drying the blocking Reagent, the slices were added with 50 μl Biotinylated anti-digoxigenin antibody and incubated at 37℃ for 60 min. After washing, the slices were added with 50 μl SABC-AP and incubated at 37℃ for 60 min. After staining with BCIP/NBT, the nuclei were re-stained with neutral red and fixed on the slide for microscopic examination [21].

### Steroid hormone analysis

The enzyme-linked immunosorbent assay (ELISA) kits (Elabscience Biotechnology Co., Ltd.) were used to determine the concentrations of testosterone in mouse serum, according to the manufacturer’s instructions. The detection ranges of testosterone was from 0.31 to 20 ng/mL respectively. Briefly, after being anesthetized with 10% sodium pentobarbital (45 mg/kg), mice were sacrificed, and trunk blood was collected and centrifuged at 1500 rmp at room temperature for 20 min. The supernatants were collected and used to measure the testosterone level of each sample. After adding 50 μL of standard working solution or sample was to each well, 50 μL of Biotinylated Detection Ab working solution were then added to each well and incubated at 37℃ for 45 min. After washing, 100 μL of HRP Conjugate working solution was added to each well and Incubated at 37℃ for 30 min. After washing, 90 μL of Substrate Reagent was added to each well and incubated at 37℃ for about 15 min. And then 50 μL of Stop Solution was added to each well. The optical density (OD value) of each well was determined by using a micro-plate reader set to 450 nm [22, 23].

### Statistical analysis

Data analyses were conducted using GraphPad Prism 8 (GraphPad Software, Inc., La Jolla, CA, USA). All data are expressed as mean ± Standard deviation (SEM). Experiments with more than two groups were analyzed using one-way ANOVA, followed by post hoc Bonferroni tests for multiple comparisons. The value of *P* < 0.05 was considered as statistically significant.

## Results

### Effects of betaine on organ and sperm parameters in chronic stressed mice

On the 3rd week, the animals were injected intraperitoneally with betaine at 200 mg/Kg (Fig. 1A). One-way ANOVA showed a significant difference among the groups in the sperm count (*F*_(2,15)_ = 18.42, *P* < 0.0001), viability rate (*F*_(2,15)_ = 19.85, *P* < 0.0001) and sperm deformity rate (*F*_(2,15)_ = 7.824, *P* = 0.00047) (Table 4). Bonferroni post-test analysis revealed that the sperm count (t = 6.058, *P* < 0.0001) and viability rate (t = 6.254, *P* < 0.0001) decreased significantly in the CRS + NS group compared with the Control + NS group, while the sperm deformity rate (t = 1.845, *P* = 0.2548) significantly increased in the CRS + NS group. BET treatment can reverse this situation (sperm count: t = 2.719, *P* = 0.0475; viability rate: t = 3.796, *P* = 0.0053; deformity rate: t = 1.845, *P* = 0.2548).

**Table 4:** Effect of Betaine (BET) on the reproductive organ weight, the reproductive organ coefficient and the sperm parameters analysis in anxiety model induced by chronic restraint stress (CRS)

Groups	Testis (mg)	Cauda Epididymis (mg)	Testis (%)	Cauda Epididymis (%)	Sperm count (10^6^ ml^−1^)	Deformity rate (%)	Viability (%)
Control + NS	107.89 ± 23.57	9.99 ± 2.18	0.39 ± 0.08	0.04 ± 0.01	8.667 ± 0.99	16.33 ± 6.41	73.56 ± 16.21
CRS + NS	97.27 ± 9.31	9.00 ± 1.29	0.39 ± 0.05	0.04 ± 0.00	4.967 ± 1.27^***^	31.33 ± 7.87^*^	33.63 ± 10.03^***^
CRS + BET	86.48 ± 36.05	10.22 ± 1.23	0.34 ± 0.15	0.04 ± 0.00	6.60 ± 0.58^#^	24.33 ± 5.16	57.86 ± 1.892^##^

^*^P < 0.05, compared with Control group; ^**^P < 0.01, compared with Control group; ^***^P<0.001, compared with Control group; ^#^*P*<0.05, compared with CRS+NS group; ^##^*P*<0.01, compared with CRS+NS group. Different symbol indicate significant difference *P* < 0.05 among groups determined by one-way ANOVA (followed by Bonferroni post hoc test)

### Betaine treatment improves the adverse effect of chronic restraint stress on the structure of testis in mice

**Fig. 2:**
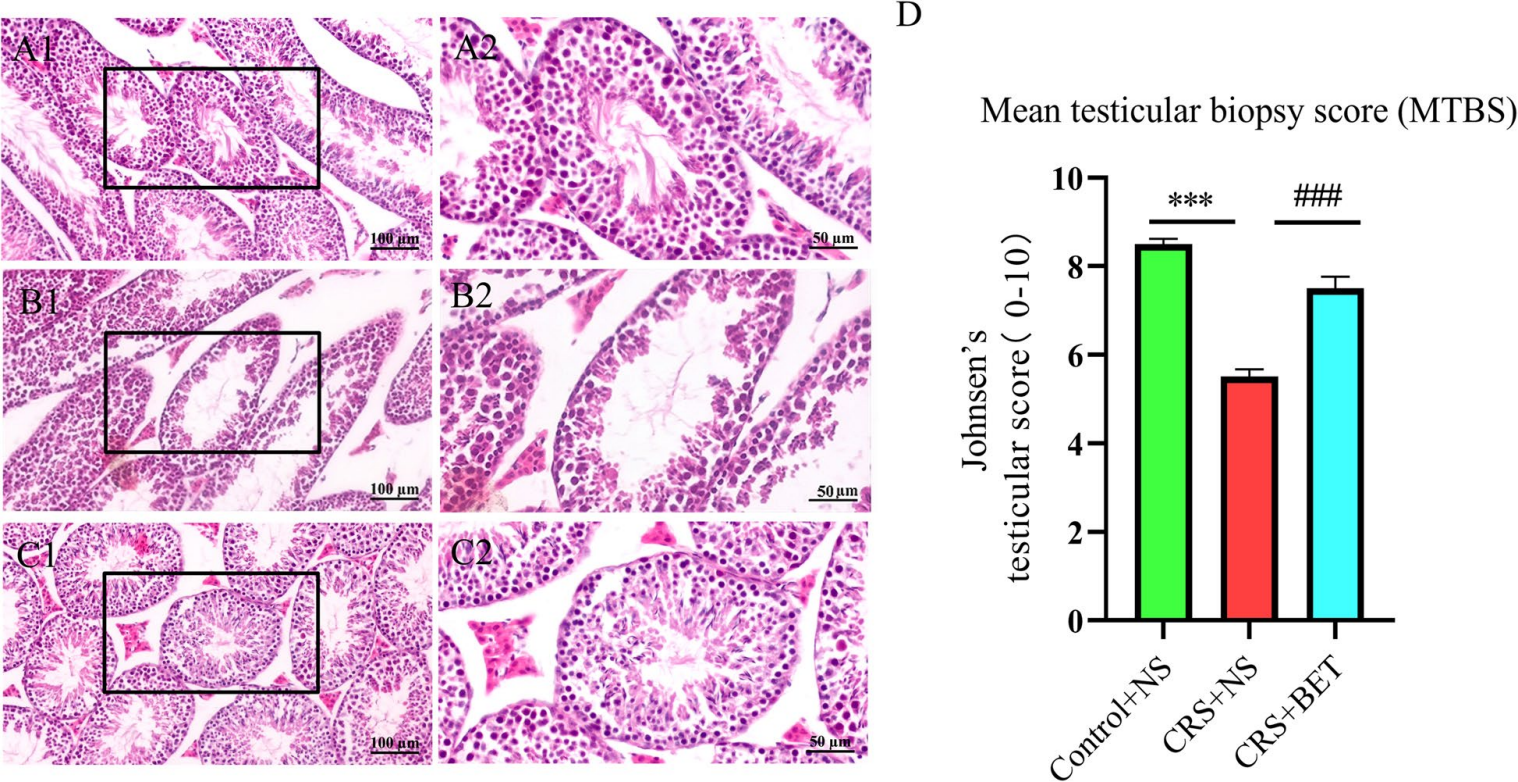
Betaine treatment improves the effect of chronic restraint stress on the structure of testis in mice. (**A1-A2**) control group; (**B1-B2**) CRS group; (**C1-C2**) BET 200 mg/ kg treated group. (**D**) Johnsen’s testicular score. Each bar indicates the mean ± SEM (*N* = 6 male mice)

The H&E results showed that the testis of CRS + NS group appeared obvious pathological structural changes, including significantly reduced diameter of the seminiferous tubules of the testes and loosely arranged spermatogenic cells, which was restored after the injection of BET. And the results were further verified by Johnsen’s scores, one-way ANOVA suggested that there were significantly differences among the groups in the Johnsen’s scores (*F*_(2,51)_ = 63.94, *P* < 0.0001). Bonferroni post-test analysis revealed that the Johnsen’s scores of CRS + NS group were significantly drop compared with the Control + NS group (t = 11.10, *P* < 0.0001), which was reversed by BET treatment (t = 7.403, *P* < 0.0001) (Fig. 2).

### Effect of betaine on spermatogenic cells in the testis of chronic stressed mice

**Fig. 3:**
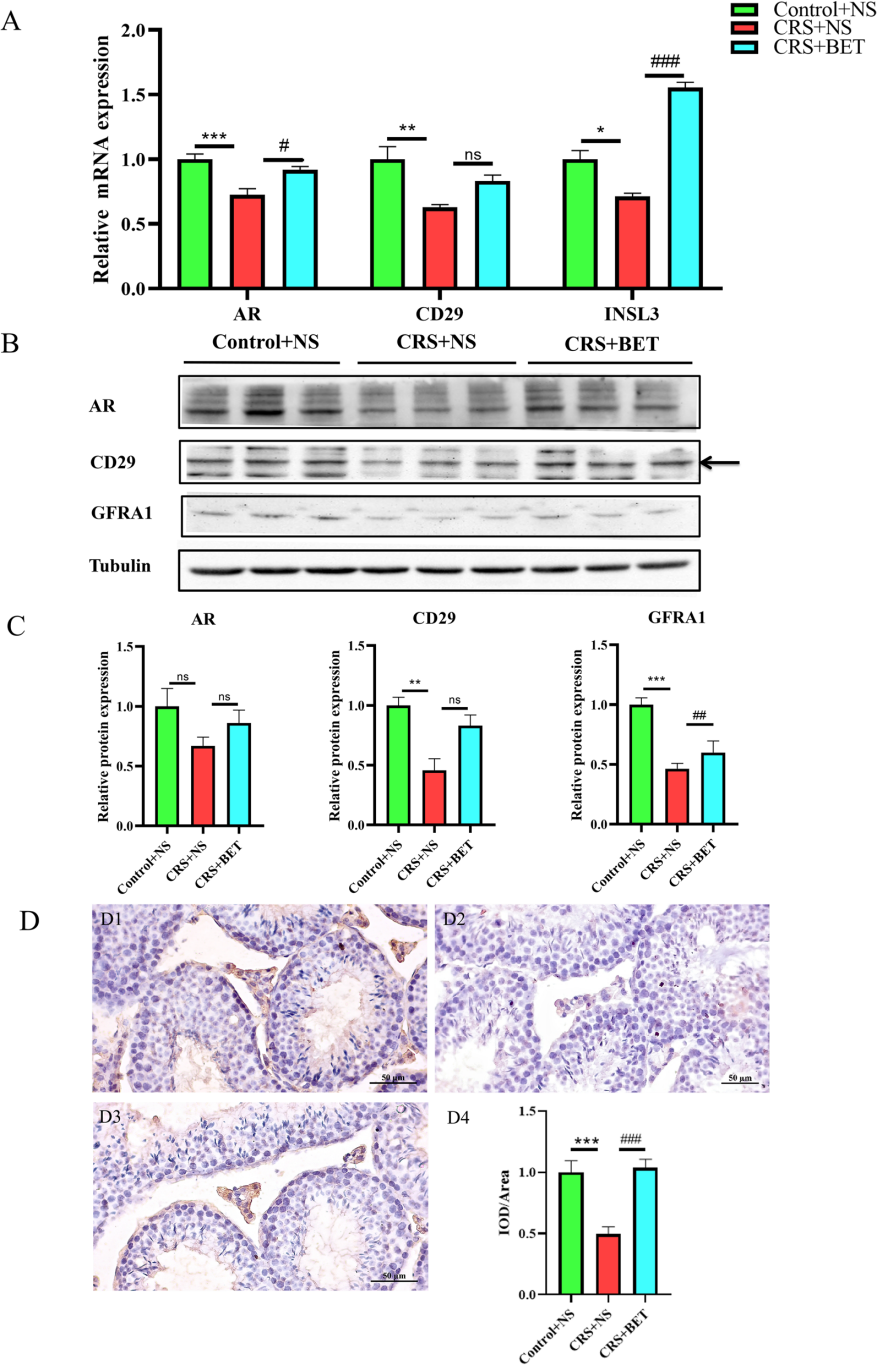
Effect of betaine on spermatogenic cells in the testis of chromic stressed mice. Each bar represents the mean ± SEM (*N* = 6 male mice). (**A**) The effect of BET on the mRNA expression level of different cell markers in the testis tissue of CRS mice; (**B-C**) Western blot analysis the effect of BET on the protein expression level of different cell markers in the testis tissue of CRS mice, Tubulin was used as the loading control; (**D**) Immunohistochemical detection of INSL3 expression in testis, (**D1**) control group; (**D2**) CRS group; (**D3**) BET 200 mg/ kg treated group (**D4**) semi-quantitative statistics; ^*^P < 0.05, compared with Control group; ^**^P < 0.01, compared with Control group; ^***^P < 0.001, compared with Control group; ^##^*P* < 0.01, compared with CRS + NS group; ^###^*P* < 0.001, compared with CRS + NS group; Different symbol indicate significant difference *P* < 0.05 among groups determined by one-way ANOVA (followed by Bonferroni post hoc test)

To further investigate the effects of betaine on various spermatogenic cells in the testes of CRS mice, the levels of Androgen receptor (AR,a marker of Sertoli cells), CD29 (a marker of spermatogonial stem cells) and INSL3 (a marker of Leydig cells) were detected by PCR (Fig. 3A). As shown in Fig. 3, there were significant differences among the three groups in the expression of spermatogenic cell markers (AR:*F*_(2,13)_ = 12.71, *P* = 0.0009; CD29:*F*_(2,13)_ = 8.301, *P* = 0.0048; INSL3:*F*_(2,15)_ = 81.94, *P* = 0.0001). Bonferroni post-test analysis revealed that the level of spermatogenic cell marker was significantly reduced in the CRS + NS group compared with Control + NS group (AR:t = 4.961, *P* = 0.0008; CD29:t = 4.070, *P* = 0.0040; INSL3:t = 4.289, *P* = 0.0019), which was reversed by BET treatment (AR:t = 3.355, *P* = 0.0155; CD29:t = 1.999, *P* = 0.2009; INSL3:t = 12.59, *P* < 0.0001).

And the results were further verified by western blot (Fig. 3B-C). There were significant differences among the three groups (AR:*F*_(2,23)_ = 1.976, *P* = 0.1615; CD29:*F*_(2,23)_ = 10.45, *P* = 0.0006; GFRA1:*F*_(2,15)_ = 16.03, *P* = 0.0002). Bonferroni post-test analysis revealed that the levels of spermatogenic cell marker was significantly reduced in the CRS + NS group compared with Control + NS group (AR:t = 1.984, *P* = 0.1780; CD29:t = 4.493, *P* = 0.0005; GFRA1:t = 5.440, *P* = 0.0002), which was recovered by BET treatment (AR:t = 1.157, *P* = 0.7773; CD29:t = 3.095, *P* = 0.0153; GFRA1:t = 1.361, *P* = 0.5811).

The results of immunohistochemistry showed that there were significant differences among the three groups in the expression of INSL3 (Fig. 3D) (F_(2, 76)_ = 14.12, *P* < 0.0001). Bonferroni post-test analysis revealed that The levels of INSL3 was significantly reduced in the CRS + NS group compared with Control + NS group (t = 4.506, *P* < 0.0001), which were recovered by BET treatment (t = 4.788, *P* < 0.0001).

Taken together, these results indicated that BET treatment led to increased expression of various spermatogenic cell in the testis of CRS mice.

### Effects of betaine on testicular apoptosis and serum testosterone levels in chronically stressed mice

**Fig. 4:**
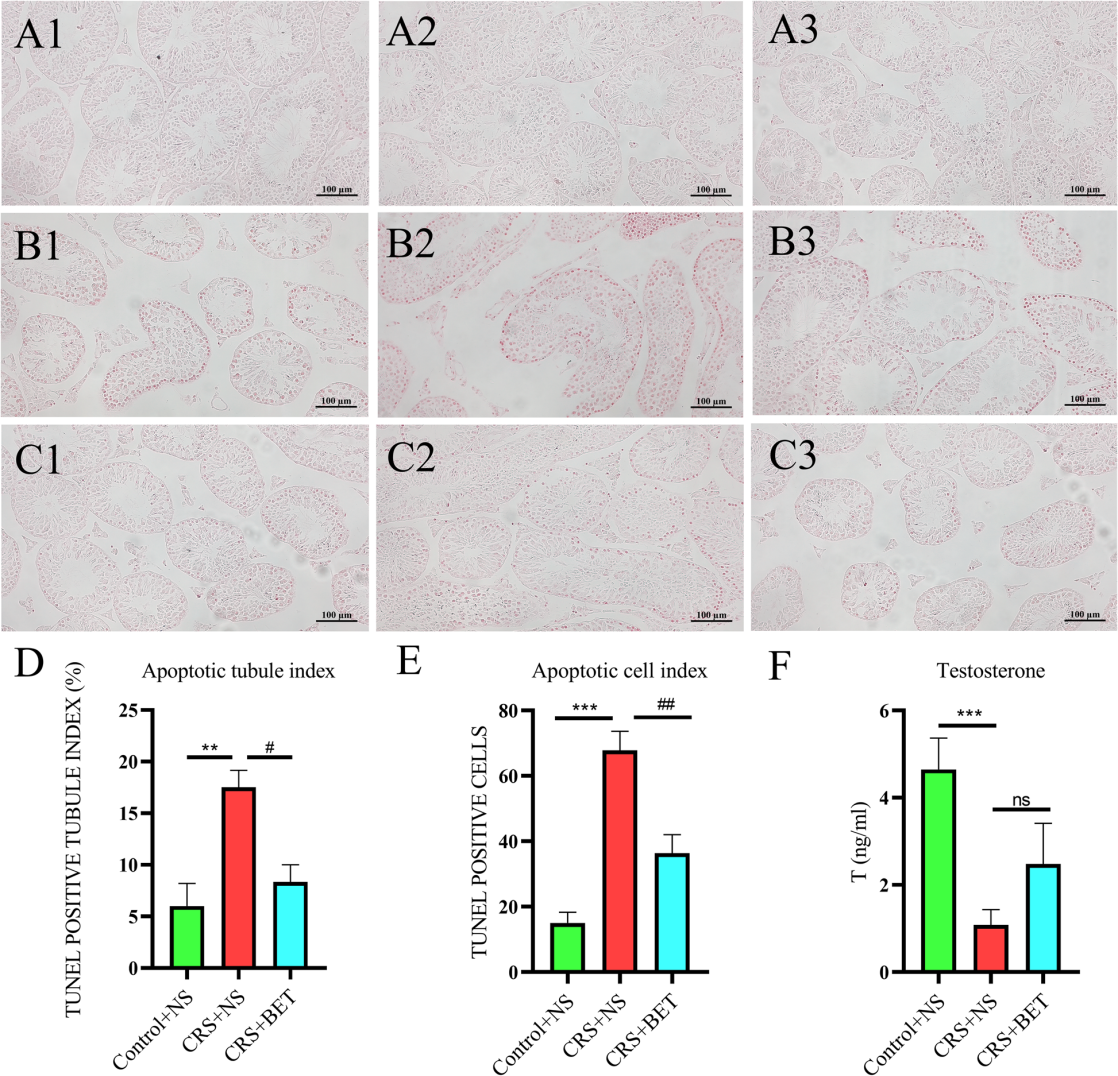
Effects of betaine on testicular apoptosis and serum testosterone levels in chronically stressed mice. (**A1-A3**) control group; (**B1-B**3) CRS group; (**C1-C3**) BET 200 mg/kg treatment group. (**D**) Apoptotic seminiferous tubule index. (**E**) Apoptotic testis cell index. (**F**) Mouse serum testosterone levels. Each bar represents the mean ± SEM (*N* = 6 male mice). ^**^P < 0.01, compared with Control group; ^***^P < 0.001, compared with Control group; ^#^*P* < 0.05, compared with CRS + NS group; ^##^*P* < 0.01, compared with CRS + NS group. Different symbol indicates significant difference *P* < 0.05 among groups determined by one-way ANOVA (followed by Bonferroni post hoc test)

Since inflammation can induce apoptosis in testicular cells, we also used TUNEL apoptosis detection kit to detect the apoptosis level of testicular cells in mice testis. As shown in Fig. 4A-C, there were significant differences among the three groups in the Apoptotic tubule index (*F*_(2,15)_ = 10.63, *P* = 0.0013) and apoptotic cell index (*F*_(2,15)_ = 27.38, *P* < 0.0001). Bonferroni post-test analysis revealed that the apoptosis levels of Apoptotic tubule index (t = 4.362, *P* = 0.0017) and apoptotic cell index (t = 7.355, *P* < 0.0001) were significantly increased in the CRS + NS group compared with Control + NS group, which was reversed by BET treatment (Apoptotic tubule index:t = 3.477, *P* = 0.0101; Apoptotic cell index:t = 4.385, *P* = 0.0016).

To understand the effect of BET on serum testosterone levels in mice, we used a testosterone (T) enzyme-linked immunosorbent assay kit to detect serum testosterone levels in peripheral blood of mice. As shown in Fig. 4F, there were significant differences among the three groups in serum testosterone levels in mice. (*F*_(2,23)_ = 6.990, *P* = 0.0042). Bonferroni post-test analysis revealed that serum testosterone levels was significantly reduced in the CRS + NS group compared with Control + NS group (t = 3.668, *P* = 0.0038), but not reversed by BET treatment (t = 1.288, *P* = 0.6317).

Thus, BET treatment can reduce the apoptosis level of testicular cells in the testis of CRS mice.

### Betaine reduces testicular oxidative stress in chronic stressed mice

**Fig. 5:**
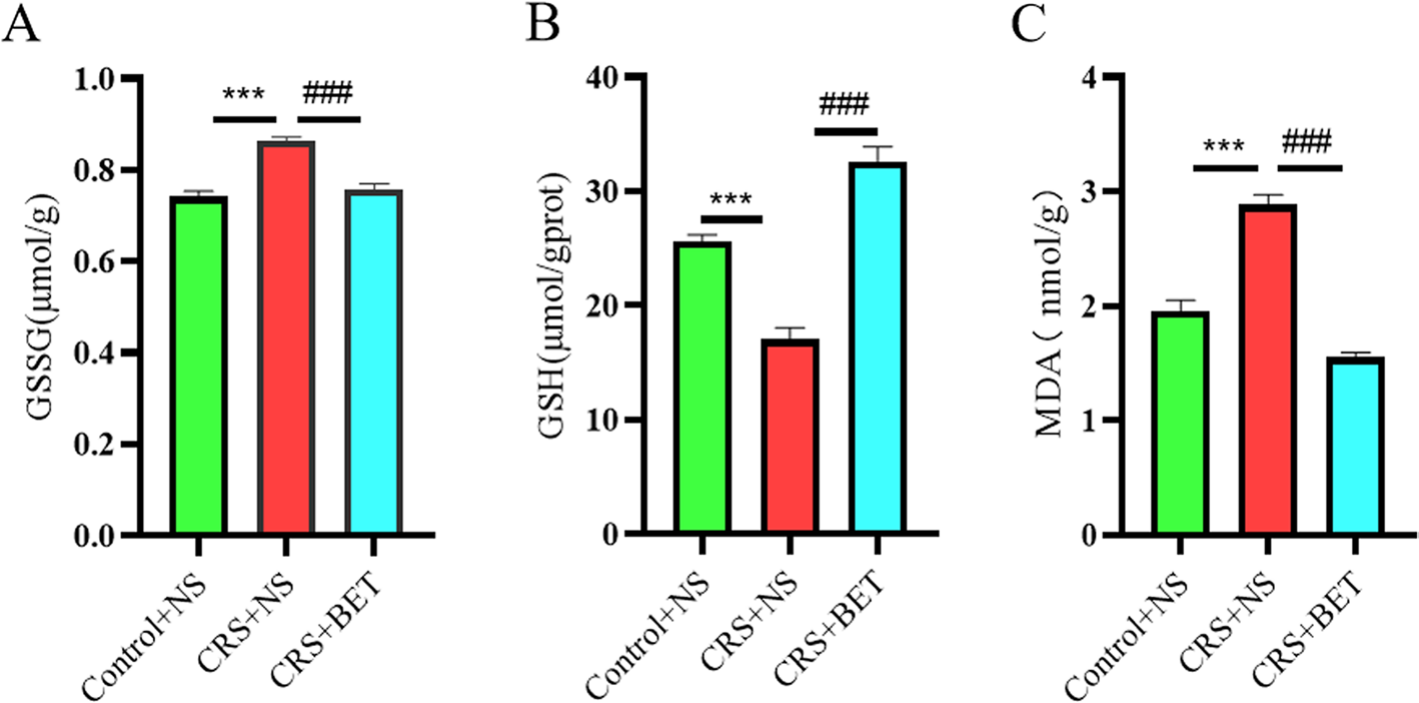
Biochemical analysis of antioxidant status. Each bar represents the mean ± SEM (N = 6 male mice). (A) Effect of BET on the levels of oxidized glutathione (GSSG) in the testis of CRS mice; (B) BET on the levels of CRS mice The effect of reduced glutathione (GSH) level in testis tissue; (C) The effect of BET on the content of malondialdehyde (MDA) in testis tissue of CRS mice; ^*^P < 0.05, compared with Control group; ^***^P < 0.001, compared with Control group; ^###^*P* < 0.001, compared with CRS + NS group; Different symbol indicate significant difference *P* < 0.05 among groups determined by one-way ANOVA (followed by Bonferroni post hoc test)

We measured testis oxidative Glutathione (GSSG) content, Reduced Glutathione (GSH) content and malondialdehyde (MDA) content (Fig. 5). As shown in Fig. 5, there were significant differences among the three groups in the levels of GSSG (*F*_(2,15)_ = 29.25, *P* < 0.0001), GSH (*F*_(2,15)_ = 60.39, *P* < 0.0001), MDA (*F*_(2,33)_ = 71.17, *P* < 0.0001). Bonferroni post-test analysis revealed that the levels of MDA (t = 8.134, *P* < 0.0001) and GSSG (t = 7.012, *P* < 0.0001) were significantly increased in the CRS + NS group compared with Control + NS group, which were reversed by BET treatment (MDA:t = 11.63, *P* < 0.0001; GSSG:t = 6.151, *P* < 0.0001). Bonferroni post-test analysis revealed that the level of GSH was significantly reduced in the CRS + NS group compared with Control + NS group (t = 6.034, *P* < 0.0001), which was revserd by BET treatment (t = 10.97, *P* < 0.0001). From the results we know that BET can prevent the over-oxidative stress in testis of CRS mice.

### Effects of betaine on the level of inflammatory cytokines in the testis of chronic stressed mice

**Fig. 6:**
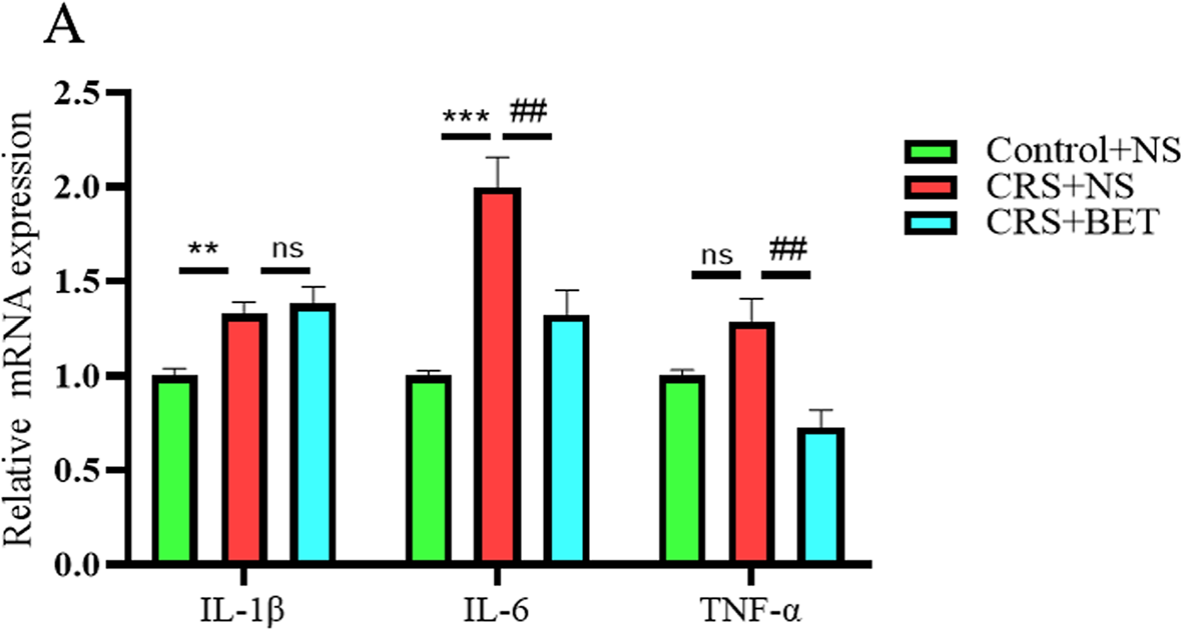
Effects of betaine on the level of inflammatory cytokines in the testis of chronic stressed mice. The effect of BET on the inflammation-related markers IL-1B, IL-6 and TNF- in the testis of CRS mice The influence of A level, each bar represents the mean ± SEM (*n* = 6 male mice); ^**^P < 0.01, compared with Control group; ^***^P < 0.001, compared with Control group; ^##^*P* < 0.01, compared with CRS + NS group; Different symbol indicate significant difference *P* < 0.05 among groups determined by one-way ANOVA (followed by Bonferroni post hoc test)

As peroxidative stress can cause inflammation in the testis, we also used PCR to detect the expression of inflammatory factors in the testis of mice. As shown in Fig. 6, significant differences was found amoung the three groups in the levels of IL-1 β(*F*_(2,11)_ = 12.49, *P* = 0.0015), IL-6(*F*_(2,13)_ = 20.46, *P* < 0.0001) and TNF-α(*F*_(2,12)_ = 10.37, *P* = 0.0024). Bonferroni post-test analysis revealed that the levels of inflammatory markers were significantly increased in the CRS + NS group compared with Control + NS group (IL-1β:t = 3.823, *P* = 0.0085; IL-6:t = 6.297, *P* < 0.0001; TNF-α:t = 2.587, *P* = 0.0713), which was reversed by BET treatment (IL-6:t = 3.823, *P* = 0.0063; TNF-α:t = 4.536, *P* = 0.0020) except IL-1β (t = 0.6004, *P* > 0.9999).

These results showed that BET treatment could prevent CRS-induced increased inflammatory cytokines in the testis.

## Discussion

This study demonstrated that BET can resist the reproductive toxicity caused by chronic stress in vivo. BET reduced the level of oxidative stress and effectively restored testicular microstructure, which destroyed by CRS. The mechanisms may involve resisting apoptosis induced by oxidative stress. BET can be regard as a prominent candidate for potential clinical use in treating stress-related testicular injury.

Stress plays a key role in alterations in various physiological responses and can even lead to various diseases, including sub-fertility or infertility in men [24]. Chronic restraint stress (CRS) is one of the most potent stress models to study the effects of physiological stress on male reproductive function [25]. In this study, CRS exposure significantly impaired the sperm quality by decreasing sperm count, motility, and live/dead ratio and increasing the percentage of morphologically abnormal sperms. The obtained results coincided with those in earlier reports demonstrating that CRS exposure decreased the sperm quality in male rodents [26]. Additionally, the CRS mice also demonstrated clear testicular damage manifested by different degenerative and necrotic morphological changes, which was further proved by Johnsen’s score. In addition, an increased number of TUNEL-positive cells were seen in the seminiferous tubules in CRS mice, demonstrating that CRS significantly increases apoptosis in testis tissue. The marked decline in sperm quality and quantity recorded in CRS exposure mice in the current investigation could be related to the depletion of testosterone, which is essential for the maintenance of normal spermatogenesis [27]. The inhibition of testicular steroidogenesis could also contribute to the suppression of testosterone biosynthesis in Leydig cells [28, 29], as evidenced by a significant down-regulation of Leydig cell related genes in the testis of CRS mice.

BET was originally named after being extracted from sugar beet molasses, having the effects of improving food flavor, improving intestinal function, and increasing antioxidant capacity [30]. However, the preventive effects of BET against adverse male reproductive parameters in mice exposed to chronic restraint stress have never been documented. Interestingly, BET co-administration effectively minimized the negative impact of CRS exposure on sperm quality through improvement of sperm parameters (sperm motility, count, viability and, morphology). Furthermore, testicular damage and increased apoptosis in testis tissue were significantly mitigated by co-administration of BET, indicating by enhanced Johnsen’s score and reduced number of TUNEL-positive cells. Thus BET could also protect the testicular tissue from the histological damage mediated by CRS. Although the testosterone levels could not be fully restored by BET treatment, it showed an enhanced trend. More importantly, the reduced expression of Leydig cell genes could be partly normalized by BET treatment. Thus we consider that concurrent administration of BET could prevent the CRS induced Leydig cell dysfunction, partly restored the serum testosterone level, and consequently enhanced the spermatogenesis.

Oxidative stress is suggested to play a key role chronic stress-induced testicular germ cell apoptosis in rodents [31]. Thus, for a better understanding the mechanism underlying the protective effect of BET on CRS induced testicular damage, we analyzed the biomarkers reflecting oxidative stress in the testis. And we found that CRS mice showed testicular oxidative stress, as indicated by elevated malondialdehyde (MDA) and total Glutathione (T-GSH)/Oxidized Glutathione (GSSG) levels, together with a marked decline reduced glutathione (GSH) levels. Notably, simultaneous treatment with BET significantly ameliorated the CRS-induced testicular oxidative damage by enhancing the activity of antioxidant enzymes and GSH concentrations and decreasing the MDA and GSSG level. These findings are in consistence with earlier reports that suggest BET has an antioxidant capacity [30]. Since oxidative stress can promote the activation of proinflammatory genes, we also determined the effect of BET on the expression of proinflammatory genes in stressed mice. And we found that administration of BET along with CRS resulted in decreased expression of IL-6 and TNF-α in the testis, hence, the protective effect could be attributed to the modulatory effects of BET on oxidative stress, inflammation, and apoptosis.

## Conclusion

In conclusion, the present study indicated that the detrimental effects of CRS on testes as evident by disrupted histoarchitecture, increased oxidative stress, inflammation and apoptosis that compromise male fertility. Further, BET can be regard as a potential drug that had potential clinical use for the treatment of stress induced testicular damages.

## Supplementary Information

**Additional file 1: Supplementary file 1.** 

**Additional file 2: Supplementary file 2.**

**Additional file 3: Supplementary file 3.** 

**Additional file 4: Supplementary file 4.** 

## Data Availability

The datasets used and/or analysed during the current study are available from the corresponding author on reasonable request.

## Abbreviations

BET: Betaine
CRS: Chronic restraint stress
NS: Normal saline
MDA: Malondialdehyde
GSSGO: Oxidized glutathione
GSH: Glutathione
WBC: White blood cell
MTBS: Mean testicular biopsy score
ARA: Androgen receptor
