# Supplementary figures and images for: Betaine attenuate chronic restraint stress-induced changes in testicular damage and oxidative stress in male mice
Source: Reprod Biol Endocrinol. 2022 May 21;20:80. doi: 10.1186/s12958-022-00949-8 (PMC9123792; doi:10.1186/s12958-022-00949-8)

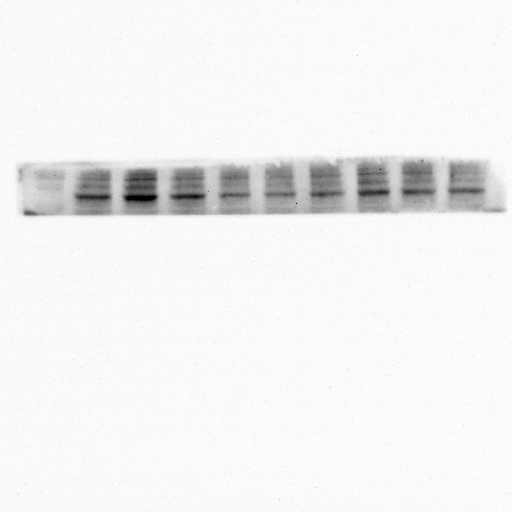

Supplement: Supplementary file 1 — Additional file 1: Supplementary file 1. [file 12958_2022_949_MOESM1_ESM.tif]

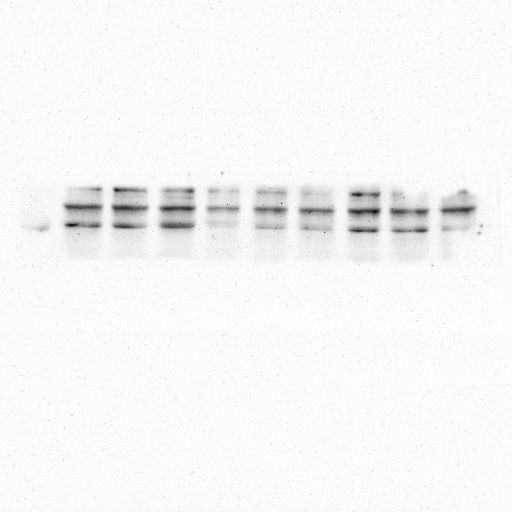

Supplement: Supplementary file 2 — Additional file 2: Supplementary file 2. [file 12958_2022_949_MOESM2_ESM.tif]

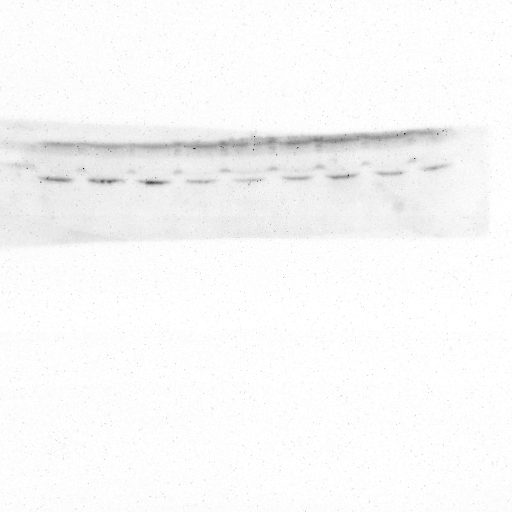

Supplement: Supplementary file 3 — Additional file 3: Supplementary file 3. [file 12958_2022_949_MOESM3_ESM.tif]

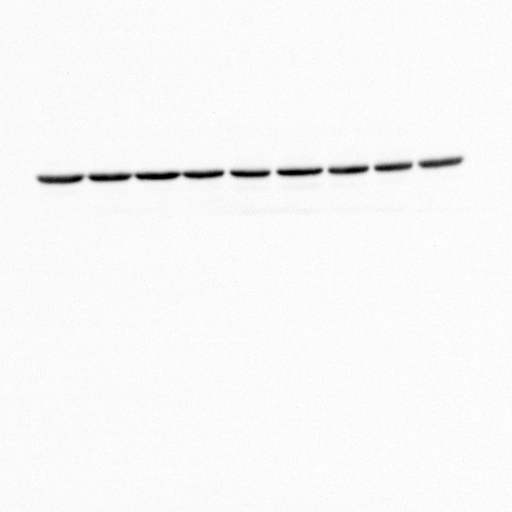

Supplement: Supplementary file 4 — Additional file 4: Supplementary file 4. [file 12958_2022_949_MOESM4_ESM.tif]
